# Shear-Enhanced Dispersion of a Wound Substance as a Candidate Mechanism for Variation Potential Transmission

**DOI:** 10.3389/fpls.2019.01393

**Published:** 2019-11-15

**Authors:** Mark G. Blyth, Richard J. Morris

**Affiliations:** ^1^School of Mathematics, University of East Anglia, Norwich, United Kingdom; ^2^Computational and Systems Biology, John Innes Centre, Norwich, United Kingdom

**Keywords:** variation potential, slow-wave potential, Ricca factor, chemical signal, electrical signaling, signal propagation, Taylor dispersion

## Abstract

A variation potential (VP) is an electrical signal unique to plants that occurs in response to wounding or flaming. The propagation mechanism itself, however, is known not to be electrical. Here we examine the hypothesis that VP transmission occurs *via* the transport of a chemical agent in the xylem. We assume the electrical signal is generated locally by the activation of an ion channel at the plasma membrane of cells adjacent to the xylem. We work on the assumption that the ion channels are triggered when the chemical concentration exceeds a threshold value. We use numerical computations to demonstrate the combined effect of advection and diffusion on chemical transport in a tube flow, and propose shear-enhanced Taylor-Aris dispersion as a candidate mechanism to explain VP rates observed in experiments.

## Introduction

A plant stem which is subjected to wounding or burning emits a slow-moving signal which can propagate long distances to remote parts of the plant. The transmission of this signal from the damage site is associated with an electrical potential waveform which can be measured experimentally and used to determine the location of the signal relative to the wound site, and hence to measure the signal’s speed and intensity. The signal itself is known as a variation potential (VP, also known as a slow wave potential), a name which refers to the change in the electrical potential on the plant surface. It travels at a rate which is on the order of 1 to 2 mm/s and is distinguished by the fact that its speed and intensity decreases with increasing distance from the wound site, and also by its ability to pass through regions of necrotic tissue (e.g. Stahlberg et al., 2006; Fromm and Lautner, 2007).

The mechanism underpinning VP transmission has been the subject of much debate, although there seems to be agreement that it cannot be electrical. To make the distinction clear between the transmission and the electrical readout that together form a VP, we refer to the propagating signal that triggers the electrical wave as the *primary signal* and the electrical wave as the *secondary signal*. The prevailing theory is that the VP is initiated by a localized, temporary increase in stem or leaf thickness which is itself induced by the passage of a high pressure wave, termed a hydraulic wave, departing from the wound site (Malone and Stanković, 1991; Stahlberg and Cosgrove, 1992; Mancuso, 1999). However, pressure waves travel relatively quickly, for example, at around 10 cm s^-1^ for wheat seedlings (Malone, 1992), and ostensibly too quickly to be the primary signal responsible for VP propagation *per se*.

Ricca (1916) proposed that the primary signal is a transported chemical agent, commonly called a wound substance or Ricca factor, which is assumed to initiate an electrical potential locally. However, the mechanism underlying this transport is less clear and common models are problematic, as reviewed by Blyth and Morris (2018). For instance, a chemical transport model based on pure diffusion provides a good fit with experimental data but only if the diffusivity is taken to be thousands of times larger than the diffusion constant in water (Vodeneev et al., 2012). A chemical transport model based purely on advection is ruled out by the viscous no-slip condition which implies that the chemical concentration at the xylem wall is zero downstream of the wound site. Evans and Morris (2017) argued that both advection and diffusion are important. They constructed an advection-diffusion transport model that included wall leakiness to provide a reasonable fit with experimental data. Despite its simplifications and approximations, this work demonstrated the plausibility of a Ricca factor as the primary signal for VP propagation.

In the present work we investigate the physical consequences of the assumption that a VP is driven by the movement of a chemical agent through the xylem. We do not attempt to describe the complex xylem architecture and approximate the xylem as a single fluid-carrying tube. We assume the presence of a preferential unidirectional fluid motion within the xylem vessels away from the wound site. This is consistent with the observation that VP signals have been observed to propagate in the opposite direction to transpiration-induced flow (root to shoot) and the hydraulic hypothesis (Mancuso, 1999) which postulates that localized damage raises the hydraulic pressure and that this may induce a flow away from the wound site. Here, we do not address the driving force for this fluid motion but, assuming fluid flow, evaluate whether the transport of a hypothetical chemical agent within the flow is consistent with experimental observations of VP propagation for the small diffusivities expected in the xylem fluid.

Our proposal is based on the theoretical approximation introduced by Taylor (1953), and later refined by Aris (1956), which shows that the combined action of advection and diffusion in a shear flow can very significantly enhance the dispersal of a chemical agent. Specifically, the effective diffusivity of the mean cross-sectional concentration in a shearing fluid motion is substantially larger than that which obtains in a quiescent fluid.

In the experiments of Vodeneev et al. (2012) the electrical activity at the stem epidermis was measured using Ag/AgCl electrodes. Different mechanisms have been proposed to explain the conversion of the propagating primary signal in the xylem to an electrical secondary signal and its transmission to the epidermis. However, as was pointed out by Evans and Morris (2017), the consequence of this transmission away from the xylem to the epidermis will be a lag time between the actual propagating signal and the electrical potential. This lag time in itself is not of central importance for the VP propagation mechanism, so in the current work we restrict ourselves to how the underlying primary signal is transmitted. We assume that a transported chemical agent in the xylem vessels triggers an electrical response *via* the activation of ion channels when the chemical binds to a surface receptor in xylem contact cells which sit adjacent to the xylem. We approximate the typical Hill-like activation of the receptor by introducing a threshold value for the concentration of the chemical agent at the surface of the xylem conduit. This mirrors ideas put forward by Vodeneev et al. (2012, 2018). Given that the conversion of the primary signal to the measured electrical secondary signal at the epidermis introduces only a lag time, this does not alter the speed of signal propagation and we can directly compare the propagation of the chemical agent with the electrical signal.

The outline of the article is as follows. First we briefly review the individual roles of advection and diffusion in chemical transport. Next we show the combined action of these two effects in a tube flow by solving the full advection-diffusion problem numerically. Finally we demonstrate that the mechanism of shear-enhanced dispersion is a strong candidate for explaining observed VP transmission rates.

## Signalling *Via* Chemical Transport

We analyze the transport of a chemical agent through the xylem, working on the assumption that the electrical signal of the VP is generated locally by the activation of an ion channel at the plasma membrane of xylem contact cells. Assuming further that these ion channels are triggered by the binding of the chemical (characterized by a threshold concentration level), this implies that a key variable is the chemical concentration at the xylem wall, meaning in the present model the boundary of the fluid conduit.

To a first approximation we neglect the geometrical complexities of the true xylem architecture and model a section as a long fluid-filled tube of circular cross-section and radius *a*. The chemical is transported by a unidirectional Poiseuille flow parallel to the tube axis that is driven by a constant axial pressure gradient. Working with respect to cylindrical polar coordinates (*x*,*r*,θ), with the tube wall located at *r* = *a*, we write this pressure gradient as d*p*/d*x* = -*G*, for constant *G* > 0, where *p* is the fluid pressure. The axial velocity component is given by [e.g. Blyth and Morris (2018)]

(1) $$u(r)=\frac{G}{4\mu }(a^{2}-r^{2}),$$

where *µ* is the dynamic viscosity of the fluid. The chemical concentration *c*(*x*,*r*,*t*) satisfies the advection-diffusion equation [e.g. Blyth and Morris (2018)]

(2) $$c_{t}+uc_{x}=D(c_{xx}+{\text{c}}_{rr}+\frac{c_{r}}{r}),$$

where a subscript denotes a partial derivative and *D* is the diffusivity of the chemical in the carrier fluid. Assuming an impermeable tube wall we set the boundary condition

(3) $$c_{r}(x,\;a,t)=0$$

and we impose the regularity condition at the pipe axis, *c* *_r_* (*x,* 0*, t*) = 0. An initial condition is also required to specify the distribution of the chemical at *t* = 0 which is itself determined by the release of the chemical into the xylem in response to wounding. This advection-diffusion problem for the chemical concentration is mathematically challenging and a solution can usually only be obtained using approximate analytical methods or by numerical computation. Even so considerable insight can be gained by studying the effects of advection and diffusion in isolation. We present a brief review in the following subsections, and in particular we discuss the standalone deficiencies of either advection or diffusion in explaining the propagation of a VP. As was noted above, a key variable of interest in this respect is the concentration of the chemical at the wall, *w*(*x*,*t*) ≡ *c*(*x*,*a*,*t*).

### Advection as the Transport Mechanism

In the absence of diffusion the transport is governed by advection alone. In this case Equation 2 reduces to the simplified form d*c*/d*t* = 0 (e.g. Blyth and Morris (2018)) so that that the convective derivative of the chemical concentration vanishes: this means that the concentration identified with an individual fluid particle does not change as the particle is carried with the flow. In the circular tube flow under consideration the trajectory of a particular fluid particle is given by

**Figure 1:**
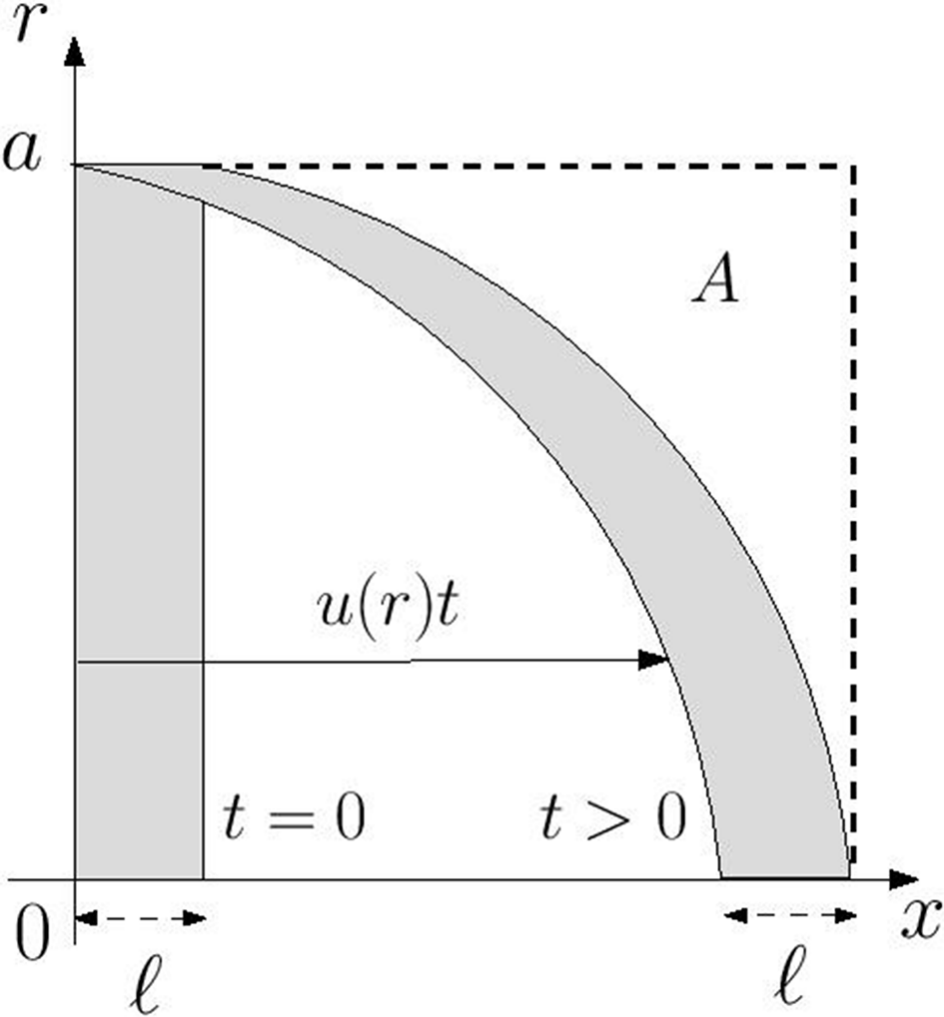
Advective distortion of an initially disk-shaped region of chemical of axial width ℓ in a Poiseuille flow with axial velocity component *u*(*r*). At any time *t*  > 0 the region marked *A*, in which ℓ + *u*(*r*)*t* ≤ *x* ≤ ℓ + *u*(0) *t*, is devoid of chemical.

(4) x(t)=G4μ(a2−r02)t+x0, r(t)=r,0

where *x*(*t*) and *r*(*t*) are the coordinates of the particle at time *t* and *x*_0_, *r*_0_ are the initial location of the particle at time *t* = 0. As time increases an initially disk-shaped region of chemical distorts into a parachute-shaped configuration as is illustrated in **Figure 1**. Notably for any time *t* > 0 there is no chemical in the region marked *A* which is defined by

(5) $$\ell +\frac{G}{4\mu }(a^{2}-r^{2})t\leq x\leq \ell +\frac{Ga^{2}}{4\mu }t.$$

At the wall the region *A* extends over the range ℓ ≤ *x* ≤ ℓ+(*Ga* ^2^/4*µ*)*t* meaning that the wall concentration at any point in this region satisfies the relation *w*(*x*,*t*) = *w*(*x*,0). Accordingly the chemical cannot reach any point on the wall downstream of the portion occupied by the initial distribution. This is indicated graphically by the distortion of the disk-shaped region in **Figure 1**. Thus, under the assumption that flow in each xylem vessel is unidirectional, advection alone is unlikely to be responsible for VP transmission.

### Diffusion as the Transport Mechanism

The one-dimensional form of the advection-diffusion equation (2) is *c* *_t_* *+* *Uc* *_x_* = *Dc* *_xx_*, where *U* is a constant. This equation has been used to model chemical transport in the xylem and, thereby, to estimate the speed of a VP. Ignoring advection (so that *U* = 0), Vodeneev et al. (2012) solved this equation to track the critical point where the concentration is just at the threshold level, σ say, required to trigger an electrical signal. Evans and Morris (2017) carried out a similar calculation but for *U* ≠ 0. In the latter case the solution takes the form

(6) $$c=\frac{C}{{\left(4\pi Dt\right)}^{1/2}}{\text{e}}^{-{\left(x-Ut\right)}^{2}/4Dt},\;C=\int_{-\infty }^{\infty }c\text{ d}x,$$

where *C* is the total mass of chemical which, we note, is independent of time. Under pure diffusion (*U* = 0) this solution represents an initial highly localized distribution of chemical which spreads out equally in both directions over time. We denote by *x* = γ(*t*) the location of the critical points at which the wall concentration is at the threshold level, that is *w* = σ. According to Equation 6,

(7) $$\gamma (t)=Ut\pm {\left[4Dt\left(\log(C/\sigma \right)-\frac{1}{2}\log(4\pi \text{D}t)\right]}^{1/2},$$

where the ± sign indicates that there are two such critical points. The plus sign denotes the leading critical point that determines when the threshold level is first exceeded at any given point on the tube wall downstream of the deposition region. The minus sign indicates the rearward critical point that lags behind, but which determines how far upstream the signal can reach along the wall (see *Numerical Computations*). Henceforth we shall use γ and γ_R_ to refer to the leading and rearward critical points, respectively. The result (7) indicates that there is a theoretical maximum distance that can be travelled by either critical point for any combination of σ and *C*. This maximum distance is attained at the time when the term inside the large curved bracket in (7) reaches zero. However, for parameter values appropriate for the xylem, this time is huge (on the order of years) and so is not a practical concern.

Vodeneev et al. (2012) showed that the result (7) with *U* = 0 provides a good fit with experimental data but only if the diffusivity *D* is taken to be about 0.045 cm^2^ s^-1^, which is approximately 2,000 times larger than the value expected for small molecules in a water solution [according to Levich (1962), p. 53, this is roughly 10^-5^–10^-6^ cm^2^ s^-1^]. Nevertheless their prediction does capture the well-known phenomenon that VP speed is retarded with propagation distance. Vodeneev et al. (2018) recently evaluated an extended version of their ‘turbulent diffusion’ model that includes the active production of the wounding substance. They nicely demonstrate how different parameters settings can recapitulate observed VP characteristics, such as propagation speed and amplitude changes with distance, for the different VP initiation treatments burning, heating and crushing. Using an advection speed *U* = 0.17 cm s^-1^, and incorporating a degree of leakiness at the tube wall, Evans and Morris (2017) obtained a reasonable fit with Vodeneev et al. (2012)'s experimental data using the more physically plausible value of the diffusivity *D* = 10^-6^ cm^2^ s^-1^ (Mastro et al., 1984).

### Numerical Computations

As we have noted, under pure advective transport the chemical cannot enter the region marked *A* in **Figure 1**. In fact whatever the initial chemical distribution the wall concentration downstream will remain zero for all time, meaning that there is no VP transmission. Diffusion acting alone requires an exorbitant value of the diffusivity to match observed VP speeds; however, diffusion does provide a mechanism to allow chemical to penetrate the empty region *A* and to reach the wall to trigger an electrical signal. In this subsection we investigate the combined action of these two effects to facilitate VP transmission by chemical transport by solving the advection-diffusion problem for the chemical concentration numerically using a finite difference alternating direction implicit (ADI) method [e.g. Hoffman (1992)].

It is numerically convenient to work in a frame of reference travelling in the positive *x* direction with the cross-sectional average of the flow velocity *ū* = *Ga* *^2^*/(8µ). In this moving reference frame the advection-diffusion problem stated in *Signalling via Chemical Transport* takes the form

(8) $$c_{t}+(u-\bar{u})c_{z}=D\left(c_{zz}+c_{rr}+\frac{c_{r}}{r}\right),$$

where *z* = *x* - *ū t* with boundary conditions *c* *_r_*(*z*,*a*,*t*) = 0 and *c* *_r_*(*z*,0,*t*) = 0. The problem is solved over a computational domain of length *L* taken to be sufficiently large so that the chemical does not reach the ends over the duration of the simulation. For definiteness, we impose the zero-flux end conditions

(9) $$Dc_{z}=(u-\bar{u})c$$

at *z* = 0,*L*. In the computation to be presented we take *L* = 25*a*. The initial condition is set as

(10) $$c(z,r,0)=\{\begin{array}{ccc} 1 & \text{if} & 9a\leq z\leq 10a,\\ 0 & & \mathrm{otherwise} \end{array}$$

**Figure 2:**
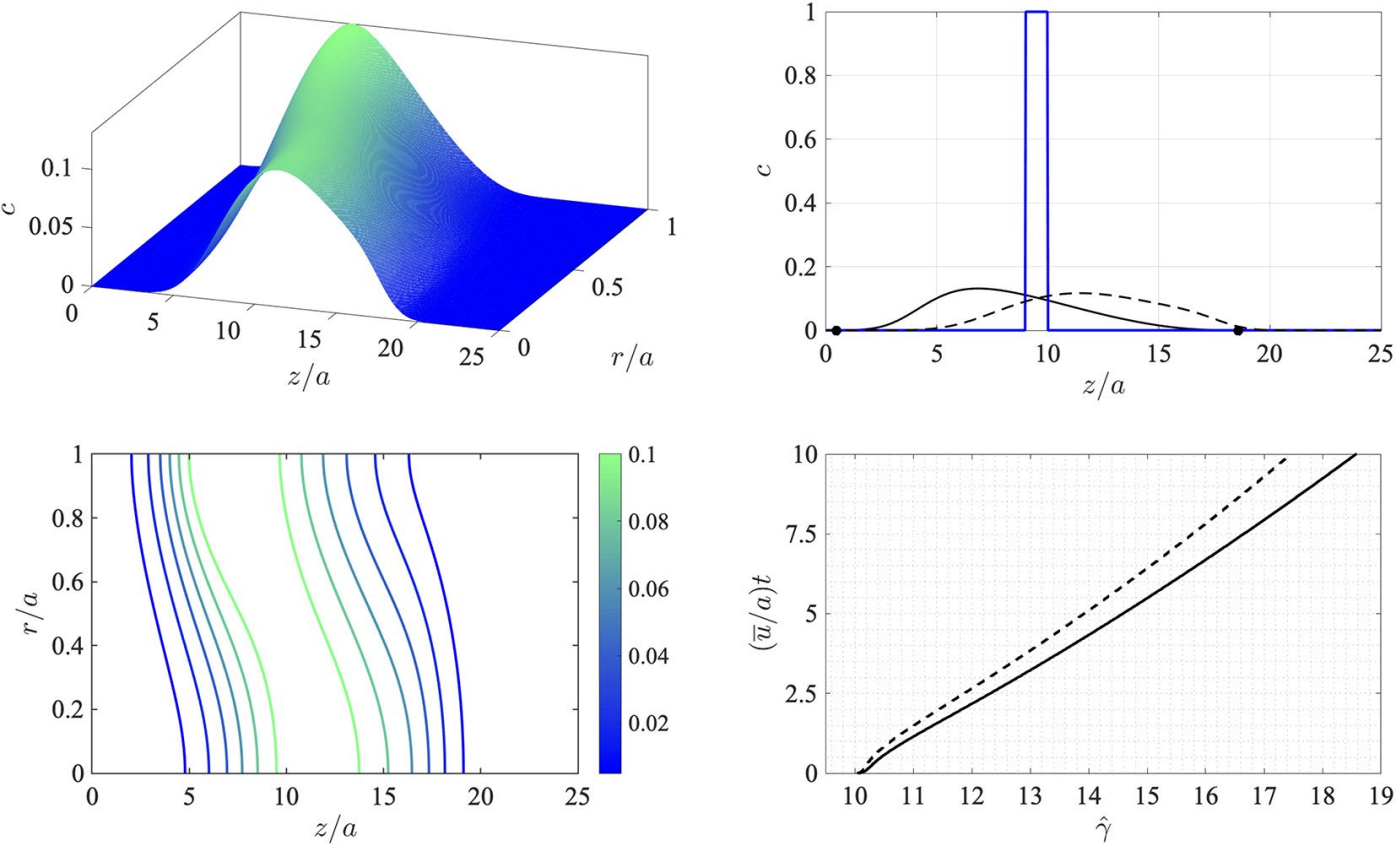
Numerical simulation of the advection-diffusion problem (8)-(10) in a reference frame moving at the average flow speed *u¯*. Top left: surface concentration plot at (*u¯*/*a*)*t* = 10. Bottom left: concentration contours at (*u¯*/*a*)*t* = 10. The Péclet number is *Pe* = *a u¯/D* = 30.0. Top right: wall concentration *c*(*z*,*a*,*t*) (solid line) and centerline concentration *c*(*z*,0,*t*) (dashed line) at time (*u¯*/*a*)*t* = 10 shown against dimensionless distance *z*/*a* (the initial condition at *t* = 0 is indicated by the thick solid line). The location of the leading and rearward critical points at (*u¯*/*a*)*t* = 10 are shown with filled circles. Bottom right: the trajectory of the downstream-moving critical point in the moving frame $\hat{\gamma }$ for the threshold concentration σ = 10^–4^ (solid line) and σ = 10^–4^ (dashed line).

This corresponds to an initial state comprising a circular disk-shaped region filled with chemical at a uniform concentration. The finite-difference approximations were computed on a uniform grid in this frame of reference with 200 equally-spaced points over 0 ≤ *r*/*a* ≤ 1 and 800 equally-spaced points over 0 ≤ *z*/*a* ≤ 25. These were deemed *via* resolution checks to be sufficiently large numbers of points to provide accurate results over the duration of the simulation. The time step was taken to be (*ū/a*)d*t* = 0.01 in dimensionless time units, and the simulation was terminated at (*ū/a*)*t* = 0.01. The problem as stated depends on two dimensionless parameters: the Péclet number *Pe* = *aū/D*, which encapsulates the relative effects of advection and diffusion, and the threshold concentration σ. Here the Péclet number was set to *Pe* = 30.0. The results are shown in **Figure 2**.

Evidently the initially sharp distribution is rapidly smeared out along the tube. Diffusion carries the chemical both upstream and downstream and also toward the wall. Consequently, and as anticipated, the region in which the wall concentration is nonzero spreads downstream in the moving frame. This is indicated by the concentration contours in the bottom left panel of **Figure 2**, which also show that the concentration level at the wall lags behind that on the tube centerline. The bottom right panel in the figure shows the trajectory of the leading critical point, given by $\hat{\gamma }=\gamma -\bar{u}t$, at which the wall concentration has reached the threshold value σ in the moving *z*-frame. After an initial transient the rate of advance of $\hat{\gamma }$ very gradually slows down (the speed of the critical point in the stationary *x*-frame therefore also slows down). Note that the value of σ makes only a minor difference to the speed of propagation as is seen by the solid and broken lines in the bottom right panel which correspond to values of σ that differ by a factor of 10.

**Figure 3:**
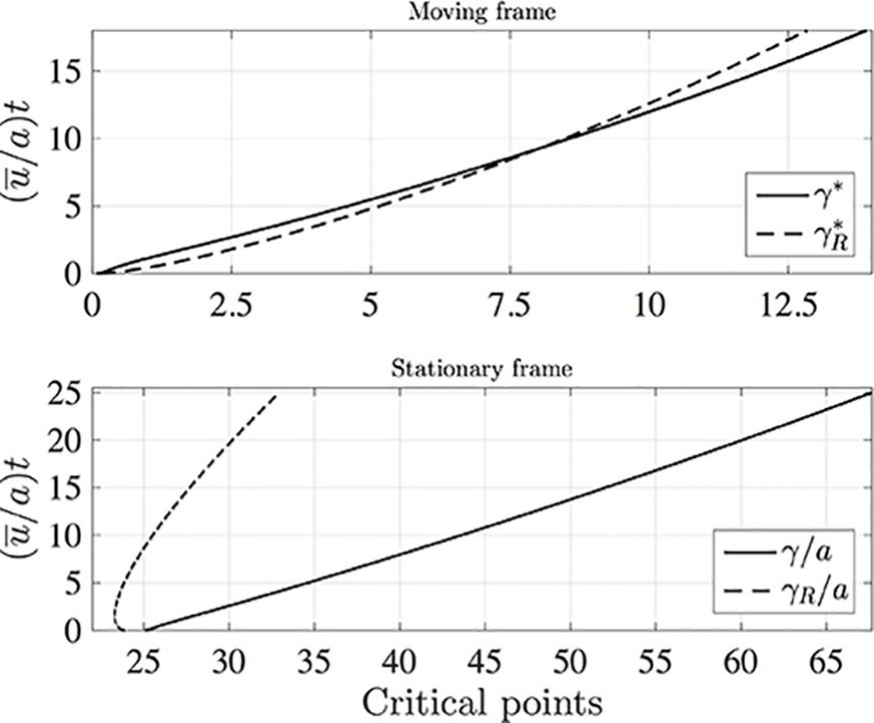
The leading (solid lines) and rearward (dashed lines) critical points for the same conditions as in Figure 2 except that in the initial condition (10) *c*(*z*,*r*,0) is non-zero in the region 24*a*≤*z*≤25*a*, and the calculation was performed in a tube of length *L*/*a* = 50 with *N* *_x_* = 1,600, *N* *_r_* = 200, and (*u¯/a*)d*t* = 0.005. Top: distances covered in the moving frame, with $\gamma^{*}=\hat{\gamma }/a-25$ and $\gamma_{R}^{*}=24-{\hat{\gamma }}_{R}/a$. Bottom: Stationary frame values γ/*a* and γ*_R_*/*a*.

**Figure 4:**
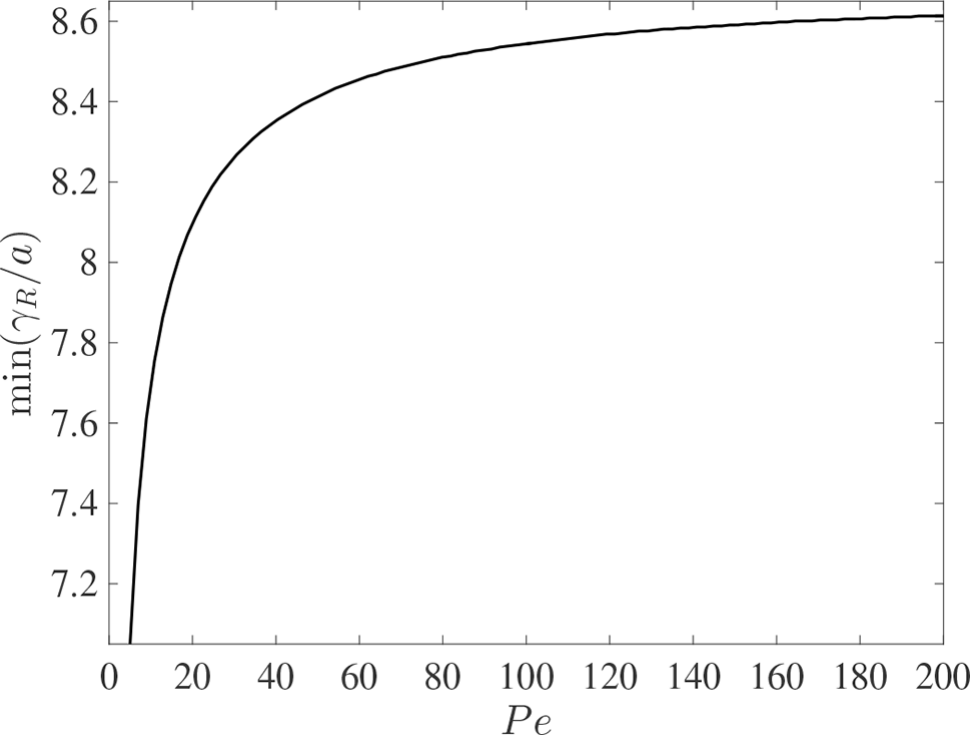
The dependence of the farthest upstream point reached by the rearward critical point, min(γ*_R_* */a*), on the Péclect number *Pe* = *au¯/D* for otherwise the same conditions as in **Figure 2** (with σ = 10^–4^). The initial condition is given in (10).

It is interesting to compare the rates at which the leading and rearward critical points progress. These are shown in **Figure 3** for the same conditions as in **Figure 2** but with the chemical mass initially concentrated in a small disk-shaped region set in the middle of a tube of twice the length in order to capture the advancing trajectories for a longer time period. The top panel shows the distances covered by each critical point in the frame of reference moving at the mean fluid velocity $\left(here\;{\hat{\gamma }}_{R}(t)=\gamma_{R}-\bar{u}t\right)$. The lower panel shows the trajectories γ/*a* and γ*_R_*/*a* in the stationary frame of the tube. Counterintuitively, the rearward point initially moves backward faster than the leading point moves forward (see the upper panel). Eventually, in the tube frame shown in the lower panel, the rearward point switches direction and starts to advance downstream. For this calculation the farthest point on the wall upstream of the deposition region that is reached by the chemical is at *x* = 8.38*a*, which corresponds to 0.62 tube radii upstream of the deposition region. **Figure 4** shows how this farthest upstream point varies with the Péclet number while holding σ = 10^–4^ constant. For low Péclect numbers diffusion dominates advection and so relatively large upstream distances are attained (formally as *Pe*→0 the chemical can reach an unlimited distance upstream as it is carried by pure diffusion in this limit). For high *Pe* advection dominates diffusion and the farthest upstream point that can be reached is much more restricted. The parameter values quoted from the literature in **Table 1** suggest some uncertainty over the value of the Péclet number in the xylem, which may be from several hundred down to about ten.

**Table 1:** Physical parameter values taken from the literature. The xylem radii quoted from Zwieniecki et al. (2001) and Malone (1996) are for one-year old ash branches and a tomato petiole, respectively.

Parameter	Units	Value (Reference)
*u¯*	cm s^-1^	0.17 (Choi et al., 2016; Evans and Morris, 2017); 0.1 (Choi et al., 2016)
*D*	cm^2^ s^-1^	10^-6^ (Levich, 1962; Evans and Morris, 2017); 10^-5^ (Levich, 1962)
σ/*C*	cm^-1^	10^–4^ (Vodeneev et al., 2012); 10^–3^ (Evans and Morris, 2017)
*a*	μm	30–60 (Zwieniecki et al., 2001); 12 (Malone, 1996)

### Advection-Diffusion as the Transport Mechanism

The numerical computations of the preceding section have shown that, working in unison, the mechanisms of advection and diffusion are able to carry a chemical agent to points on the xylem wall downstream of the wound sites and thereby to trigger an electrical signal at distal locations. However, we have also noted that an excessively large diffusivity is needed to match theoretical VP transmission rates to experimentally observed values. Evans and Morris (2017) provided an explanation for this apparent mismatch by proposing a model based on flow within a leaky tube and achieved a reasonable fit with experimental data even with a realistically small value of the diffusivity. In the Appendix we provide a theoretical justification for their leaky tube model.

An alternative explanation is provided by noting that chemical transport by both advection and diffusion in a laminar flow may be substantially enhanced in the presence of shear. In the current model the presence of shear is indicated by the radial dependence of the fluid velocity (see Equation 1). Taylor (1953), and subsequently Aris (1956), showed that the effect of shear can yield an effective diffusivity which is considerably larger than that which obtains for the same chemical agent in a stationary fluid. Under certain conditions to be stated below, the Taylor-Aris theory shows that the cross-sectional mean concentration, $\bar{c}(x,t)=(2/a^{2})\int_{0}^{a}c\;r\text{ d}r,$ satisfies the approximate equation

(11) $${\bar{c}}_{t}+\bar{u}\;{\bar{c}}_{x}=D_{e}{\bar{c}}_{xx},$$

where *ū* is the cross-sectional average of the velocity introduced earlier, and *D* *_e_* is the effective diffusivity given by

(12) $$D_{e}=D+\frac{{\bar{u}}^{2}a^{2}}{48D}$$

(see, for example, Blyth and Morris (2018)for details of the derivation of this approximation). Bailey and Gogarty (1962)showed that in practice the approximation is good for *t* greater than about 0.5*t* *_D_*, where the radial diffusion time *t* *_D_* = *a* *^2^* */D*. Formally the approximation is valid for a long tube, δ ≪ 1, provided that *t* ≫ *t* *_D_* and δ *Pe* ≪ 1, where δ = *a*/*L* is the tube slenderness parameter and *Pe* = *ūa/D* is the Péclet number. The latter two conditions stipulate that sufficient time has elapsed for the initial chemical deposit to have diffused a distance equal to one tube radius so that the concentration in a tube cross-section is almost everywhere equal to its cross-sectional mean value, and that the time taken for this cross-sectional evening-out to occur is much shorter than the timescale over which noticeable effects due to advection are observed.

To investigate whether these conditions hold in the present case, using typical parameter values from the literature (see **Table 1**), we take *D* = 10^-6^ cm^2^ s^-1^ and *a*≈30 µm to compute *t* *_D_* = 9.0 s. With *ū*≈0.17 cm s^-1^, we find that the theory should be valid after the chemical has been carried a distance of approximately 0.5 *ūt* *_D_* = 0.77 cm. This is certainly much shorter than the distances travelled in the experiments of Vodeneev et al. (2012) which are on the order of about 10 cm. Furthermore, the tracheary vessels in the xylem are long and thin and so it is reasonable to assume that δ is small. Taking *L* = 10 cm we compute δ = 3 × 10^–4^ and δ*Pe* = 0.15. We can therefore reasonably expect the aforementioned conditions on the theory to be fulfilled.

A central point of fundamental importance to the current work is that, according to (12), small values of the diffusivity *D* can lead to substantially larger values of the effective diffusivity *D* *_e_*. Taking the average of the velocity component (1) over the tube cross-section we find $\bar{u}=(2/a^{2})\int_{0}^{a}r\;u\text{ d}r=Ga^{2}/(8\mu )$. Since the effective advection speed, *ū*, in (11) is constant, we may invoke formula (7) for the location of the leading critical point at which the wall concentration *w* attains the threshold value σ given a total chemical mass *C* (see Equation 6). This gives

(13) $$\gamma =\bar{u}t+{\left[4D_{e}t\left(\log(C/\sigma )-\frac{1}{2}\log(4\pi D_{e}t)\right)\right]}^{1/2}$$

The rate of propagation of this critical point, namely γ*_t_*, and hence the rate of propagation of the VP is of particular interest. For small time γ*_t_* ≈ (*D* *_e_*/2)^1/2^(–log*t*)^1/2^/*t* ^1/2^ so that, formally, γ*_t_*→ ∞ as *t*→0. In practice, therefore, we would expect the movement of the critical point, and hence the VP, to be very rapid in the very early stages. The speed γ*_t_* decreases monotonically for *t* > 0 and will continue to slow down as time progresses. Therefore, according to this model, the VP propagation speed would continually decrease in line with the established consensus [e.g. Fromm and Lautner (2007)]. Furthermore, if we assume a link between the local wall concentration of the chemical and the strength of the electrical signal which is triggered (Sukhov et al., 2013), so that a stronger concentration implies a stronger signal, then we would also expect a reduction in the magnitude of the electrical signal which is also in line with prevailing theory.

**Figure 5:**
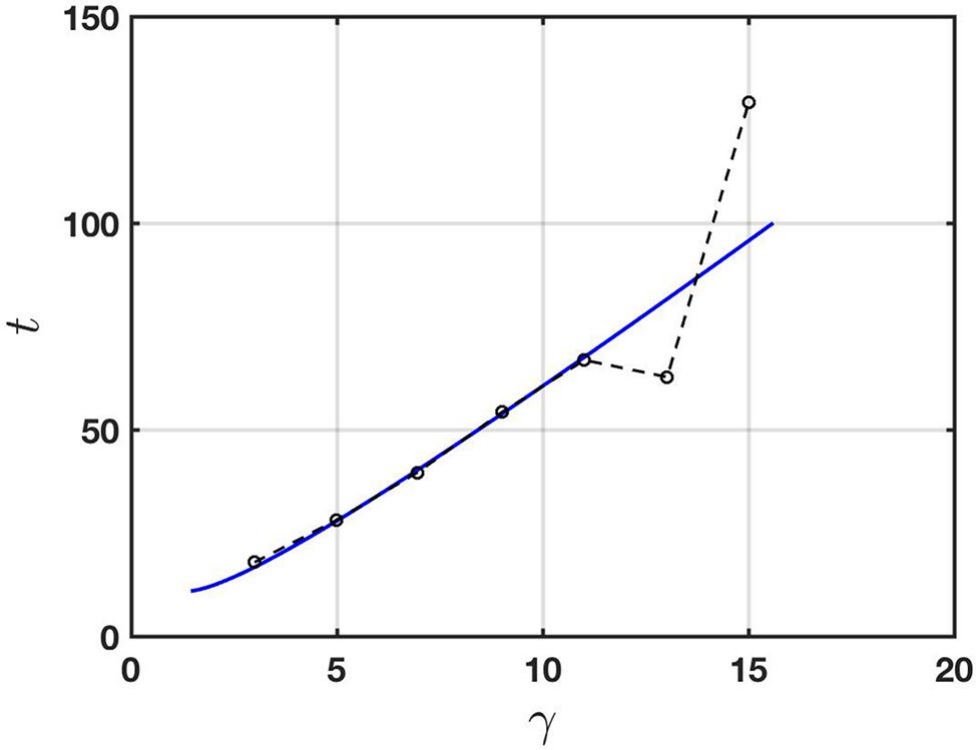
Comparison of the Taylor-Aris theory (solid line) with the experimental data of Vodeneev et al. (2012) (broken line; data points are circles). The physical parameters used for the theory are *a* = 60 μm, *u¯* = 0.12 cm s^-1^, *D* = 0.25 × 10^-5^ cm^2^ s^-1^,and σ/*C* = 0.0001 cm^-1^. In this case the effective diffusivity according to (12) is *D* *_e_* = 0.0043 cm^2^ s^-1^.

To demonstrate consistency of the Taylor-Aris theory with physical observations, in **Figure 5** we show a comparison between the prediction (13) and the experimental data of Vodeneev et al. (2012). The physical parameters used to compute the theoretical prediction (shown as a solid line in the figure) are given in the figure caption. They all lie in the respective expected physical ranges (see, for example, **Table 1**) and were chosen to provide a best fit with the experimental data. Specifically, the solid line corresponds to formula (13) with the parameters set as given and *t* replaced by *t* = 0.8*t* *_D_* (so that the transport according to (11) is taken to effectively start at *t* = 0.8*t* *_D_* with a total mass of chemical equal to that at *t* = 0). Accordingly the solid line in **Figure 5** starts at a point in time at which the Taylor-Aris approximation is expected to be valid.

It is important to point out that whilst Evans and Morris (2017) suggested advection and diffusion as a transport mechanism, their approximation excludes the possibility of shear-enhanced dispersion. This can be seen by noting that their one-dimensional transport equation, namely *c* *_t_* *+* *Uc* *_x_* = *Dc* *_xx_* includes a constant rate of advection which may be removed *via* a Galilean transformation. This effectively reduces it to the diffusion equation in a frame of reference travelling at constant speed *U*. The spatial dependence of the advection is crucial to shear-enhanced transport.

## Discussion

We have examined the hypothesis that the propagation of a VP is made possible by the transport of a chemical agent through the xylem. We have discussed the individual roles of advection and diffusion for this process and reinforced the shortcomings of each as standalone candidate mechanisms for explaining the propagation of VPs. We have discussed the enhanced diffusion afforded by the combined action of advection and diffusion *via* Taylor-Aris theory. Our discussion has been based on the assumption that an electrical signal (*secondary signal*) is initiated *via* the activation of ion channels at the plasma membrane of xylem contact cells adjacent to the xylem. The activation is triggered when the local xylem wall concentration of a chemical agent or wound substance (*primary signal*) produced at the site of injury or stimulus exceeds a threshold level. For advection alone, the wall concentration of wound substance downstream or upstream of the wound site is precisely zero, so that the threshold can never be exceeded. Given typical diffusivities of small molecules, diffusion alone cannot account for the propagation rates observed in experiments. Here we have demonstrated that for realistic parameter values the predictions based on transport *via* advection-diffusion are consistent with available experimental data.

The nature of the chemical agent remains unknown. Reactive oxygen species (ROS) have been suggested as potential wounding substances that could propagate VPs (Vodeneev et al., 2015). ROS responses have been observed for several different stresses (Waszczak et al., 2018) and have been linked to electrical signaling (Gilroy et al., 2016). Intercellular lifetimes for ROS vary from nanoseconds to seconds, depending on the ROS species and the availability of ROS scavengers (Waszczak et al., 2018). Although these values may vary significantly in the xylem, ROS stability will make long-distance diffusion or transport unlikely. Yet, ROS are known to be involved in long-distance signaling (Gilroy et al., 2016), with ROS-induced ROS release emerging as an important propagation mechanism (Zandalinas and Mittler, 2018), often coupled with calcium waves (Evans et al., 2016) and electrical signals (Choi et al., 2016; Gilroy et al., 2016; Sukhov et al., 2019). According to these models, ROS propagates by an active, self-propagating mechanism. Whilst evidence from several treatments that block VP transmission by metabolically inhibiting cells argues against self-propagation as the main mechanism (Vodeneev et al., 2015), it is possible the active release of wound substance may contribute for certain stimuli (Vodeneev et al., 2018).

Recent observations for wounding and herbivory provide evidence for GLUTAMATE RECEPTOR-LIKE (GLR) genes as candidates for the hypothetical ion channel in our model. Furthermore, this indicates that the transport of glutamate through the vasculature may be responsible for long distance signal transmission and the initiation of wound-induced calcium waves (Nguyen et al., 2018; Toyota et al., 2018). Associated calcium-permeable channels, formed by GLR genes, have been localized to the phloem (Toyota et al., 2018) and to xylem and phloem (Nguyen et al., 2018). The localization of GLR genes with a demonstrated role in VPs suggests that both phloem and xylem cells participate in the electrical signal generation associated with VPs. Interestingly, this observation coupled with experiments using single and double glutamate receptor mutants led to the conclusion that a xylem stream transported Ricca factor is untenable for leaf to leaf VP transmission in Arabidopsis (Nguyen et al., 2018). Further work will be required to determine exactly which genes influence primary and secondary signal propagation and whether the mechanisms discussed here also trigger such wound-induced calcium waves. If so, this would suggest glutamate as a prime candidate for the Ricca factor (Ricca, 1916).

Although Taylor-Aris dispersion offers an explanation for the propagation of VPs, the causation for the underlying advection remains unclear. Plausible mechanisms might include the mass flow induced by ruptured cells at the wound site (Malone, 1993). Consistent with this is the result reported by Vodeneev et al. (2012) that the propagation of radioactive sucrose in a leaf tip was substantially increased by wounding, although Vodeneev et al. attributed the faster propagation to an enhanced diffusion coefficient resulting from turbulent flow. We note that whilst Evans and Morris, (2017) suggest that turbulent flow seems highly unlikely based on the estimated Reynolds number of 5 × 10^-2^, the enhanced diffusion postulated by Vodeneev et al. (2012) may be a consequence of Taylor-Aris dispersion as demonstrated here. Although it is important to note that Taylor-Aris dispersion is very different mechanism. Other possibilities for advection include an osmotic pressure difference established in the presence of a chemical gradient, or mass flow induced by the passage of a pressure wave through the vasculature with its origin at the wound site. Further investigations, both experimental and theoretical, are required to untangle the details of VP transmission. In particular, exciting recent experimental evidence (Nguyen et al., 2018; Toyota et al. 2018) suggests a link between VPs and potentially self-propagating calcium signals *via* GLRs in both phloem and xylem and motivating a more holistic modeling approach that includes signal transmission and electrical signal generation (Vodeneev et al., 2018). It is possible that quite different characteristics of self-propagating VP signals may be observed for different stimuli and different tissues, for example, a roughly constant self-propagating signal velocity rather than one that decreases appreciably with distance (Vodeneev et al., 2018). Vodeneev et al. (2018) constructed a mathematical model to explain this that includes active production of the wound substance. The model presented in the present work can be extended to include the effect of active production, and this is left as an avenue for future investigation.

## Author Contributions

Concept and design: MB and RM. Mathematical modeling and calculations: MB. MB prepared the figures and wrote the manuscript with contributions from RM.

## Funding

RM acknowledges support from BBSRC’s Institute Strategic Programme on Biotic Interactions underpinning Crop Productivity (BB/J004553/1) and Plant Health (BB/P012574/1).

## Conflict of Interest

The authors declare that the research was conducted in the absence of any commercial or financial relationships that could be construed as a potential conflict of interest.

## Appendix: Leaky Tube Model

In this appendix we discuss the condition under which the leaky tube calculation carried out by Evans and Morris (2017) is valid. Assuming that the Reynolds number in the xylem is small, the fluid flow is governed by the Stokes equations, which we write in the form [e.g. Blyth and Morris (2018)]

(14)
$$\begin{array}{ccc} 0 & = & -{\tilde{p}}_{X}+({\tilde{u}}_{RR}+{\tilde{u}}_{R}/R+\delta^{2}{\tilde{u}}_{XX}),\\ 0 & = & -\delta^{-2}{\tilde{p}}_{R}+({\tilde{v}}_{RR}+{\tilde{v}}_{R}/R-\tilde{v}/R^{2}+\delta^{2}{\tilde{v}}_{XX}),\\ 0 & = & \delta {\tilde{u}}_{X}+{\tilde{v}}_{R}+\tilde{v}/R, \end{array}$$

where $\left(\tilde{u},\;\tilde{v})=(\mu /a^{2}G)(u,\;v\right)$ are the scaled velocity components in the axial and radial directions respectively, $\tilde{p}=(\delta^{2}/LG)p$ is the scaled pressure, and *X* = *x*/*L* and *R* = *r*/*a*. Here *L* is a suitable axial length scale (for example, the length of the xylem), *a* is the xylem radius, δ = *a*/*L*, and µ, *G* are the dynamic viscosity of the fluid and the pressure gradient driving the flow, respectively. The leakage through the xylem wall is modeled by assuming that the radial velocity at the wall is related to the pressure difference across the wall *via* Starling’s law,

(15) $$\tilde{u}(R=1)=\delta k^{2}(P-{\tilde{p}}_{0}),$$

where $P=\tilde{p}(R=1)$, *p*_0_ is the (scaled) pressure outside the xylem, and *k* ^2^ is a constant related to the permeability of the wall. We note that in this model the local pressure affects the transport of the chemical directly *via* condition (15).

Proceeding on the basis that δ is small, so that there is only a weak rate of fluid loss through the wall, we deduce from the second equation in (14) that $\tilde{p}=\tilde{p}\;(X)$ to leading order approximation. Ignoring contributions of *O*(δ^2^) and integrating the first equation in (14) twice with respect to *R*, we obtain

(16) $$\tilde{u}=-\frac{1}{4}{\tilde{p}}_{X}(1-R^{2}),$$

where we have assumed no slip at the xylem wall. On integrating the third equation in (14) we find that the boundary condition (15) is satisfied only if the pressure takes the form

(17) $$\tilde{p}(X)={\tilde{p}}_{0}+2k^{-1}{\tilde{u}}_{00}{\text{e}}^{-2kX},$$

where *ũ*_00_ = *ũ* (*R* = 0, *X* = 0). Restoring the variable dimensions, it follows from (16), (17) that the axial velocity component at the tube axis is

(18) $$u(r=0)=u_{00}{\text{e}}^{-2\beta x/a},$$

where *β* = δ*k* and *u*_00_ is the axial velocity component on the axis at the tube entrance *x* = 0. Equation (18) is the form adopted by Evans and Morris (2017) (for comparison purposes, note that *u*_00_ is twice the cross- sectional average axial velocity at *x* = 0).

Evans and Morris adopted a one-dimensional viewpoint for the chemical transport, working with the advection-diffusion equation (2) with *u* replaced by (18). In this case the solution given by these authors (and also given here by equation (6) with *U* replaced by (18) is valid to leading order approximation in β. We conclude that the leaky tube calculation performed by Evans and Morris is valid as a first approximation provided that *β* is taken to be sufficiently small. In fact Evans and Morris (2017) used the small value *β* = 0.038 to obtain their fit.
